# Radon (^222^Rn) in underground drinking water supplies of the Southern Greater Poland Region

**DOI:** 10.1007/s10967-013-2912-1

**Published:** 2014-01-07

**Authors:** Henryk Bem, Urszula Plota, Marta Staniszewska, Ewa Maria Bem, Daria Mazurek

**Affiliations:** Higher Vocational State School in Kalisz, ul. Nowy Świat 4, 62-800 Kalisz, Poland

**Keywords:** Radon in drinking water, Liquid scintillation, Effective doses from ingestion and inhalation

## Abstract

Activity concentration of the ^222^Rn radionuclide was determined in drinking water samples from the Sothern Greater Poland region by liquid scintillation technique. The measured values ranged from 0.42 to 10.52 Bq/dm^3^ with the geometric mean value of 1.92 Bq/dm^3^. The calculated average annual effective doses from ingestion with water and inhalation of this radionuclide escaping from water were 1.15 and 11.8 μSv, respectively. Therefore, it should be underlined that, generally, it’s not the ingestion of natural radionuclides with water but inhalation of the radon escaping from water which is a substantial part of the radiological hazard due to the presence of the natural radionuclides from the uranium and thorium series in the drinking water.

## Introduction

Radon is a naturally occurring gaseous radioactive element found in most groundwater. Thanks to its fairly solubility in water it comes to the water from the decay of radium in soil or rocks adjacent to these reservoirs. There are three naturally occurring radon nuclides, but the use of the term *radon* generally refers specifically to the most important isotope ^222^Rn with half-life of 3.825 days. Radon is known to present a risk of lung cancer when it, or rather its decay products, are inhaled [1, 2] As for other radionuclides, there are two ways of radon exposure for members of public: inhalation of the indoor radon and ingestion with food and drinking water. Most of the radon that enters into a indoor air comes directly from soil and this radionuclide can there accumulate to the higher concentrations above 100 Bq/m^3^. Radon present in well water will also enter a home whenever this water is used. In many situations such as showering, washing clothes or boiling water, radon is released from the water into the indoor air. Thus, radon present in water, besides of its health hazard via direct ingestion, can also contribute to the total inhalation risk associated with its transferring into indoor air. Although the radiation risk from radon exposure through ingestion of drinking-water is much smaller of that caused by indoor inhalation of radon, many international organizations introduced some regulations concerning permissible concentrations of this radionuclide in drinking water.

The World Health Organization (WHO) guidelines for drinking water quality are based on the assumption that in the case of radionuclide ingestion over extended periods of time the resulting an effective dose rate should not exceed of 0.1 mSv/year. On the base of daily water intake of 2 dm^3^/day and dose conversion factors for particular radionuclides it was possible to determine so called guidance levels for almost all natural and anthropogenic radionuclide concentrations in drinking water. However, there is no guidance level for ^222^Rn radionuclide but only suggestion that repeated measurements should be implemented, if radon activity concentration in public drinking water supplies exceeds 100 Bq/l [3]. Similar approach has been proposed in the EU (European Union) commission recommendations: no remedial action should be required if the concentration of radon in drinking water is <100 Bq/l [4]. Therefore, seven European countries (Denmark, Finland, Germany, Greece, Ireland, Sweden and the Czech Republic) have set their own reference levels in the range 20–1,000 Bq/l for radon in drinking water [5]. The US Environmental Protection Agency (EPA) proposed in 1991 an maximum contamination level (MCL) for radon of 11 Bq/dm^3^ (about 300 pCi/dm^3^) in drinking water. However, from practical reasons now EPA recommends also another an alternative maximum contamination level (AMCL). According to EPA the AMCL is the concentration of radon in water that would cause an increase of radon in indoor air that is no greater than the level of radon naturally present in outdoor air. The average outdoor air concentration over the entire United States is about 15 Bq/m^3^ or 0.4 pCi/dm^3^. From the other side, the contribution to radon concentration in indoor air from household usage of water is very low, so called water to air average transfer coefficient-*T* is in the order of 10^−4^ [6]. After combining these data, EPA has determined that the AMCL for radon in drinking water should be about 150 Bq/dm^3^ or 4,000 pCi/dm^3^ but EPA also strongly encourages States to reconsider a higher AMCL with accompanying a multimedia mitigation (MMM) program to address radon risk on indoor radon [7].

However, the latest proposed value of AMCL equal to 150 Bq/dm^3^ for radon in water needs some comments. It corresponds to health hazard comparable with inhalation of indoor radon in concentration of 15 Bq/m^3^. According to the latest evaluation of the radon effective dose (dosimetric) coefficient by Harrison and Marsh this value is equal to 22 nSv per Bq h m^−3^ [8]. It means that annual effective dose corresponding to inhalation of radon in concentration of 15 Bq/m^3^ will be equal to 0.9 mSv (assuming 7,000 h per year indoor occupancy and equilibrium factor with its progeny—*F* = 0.4). This value also corresponds to the effective dose from domestic usage of water with radon in concentration of 150 Bq/dm^3^, excluding internal dose from its intake Therefore, the total effective dose from this source of exposure is at least nine times higher than WHO’s individual dose criterion (IDC) of 0.1 mSv/year for all radionuclides in drinking water.

Although that range of an effective radiation doses ~1 mSv is only fraction of the total average annual exposure of humans from all radiation sources equal to 3.3 mSv, the recent evidence on the risks of very low-level radiation supports the LNT (linear no-threshold) model and indicates harmful radiation effects well below 100 mSv. Particularly, a well statistically based epidemiological studies indicate adverse effects to people exposed to very low doses ~10 mSv: from medical CT (computer tomography) scans to infants [9], to Chernobyl clean-up workers [10] and even reveal adverse effects from background radiation to which all of us are exposed [11]. Therefore, each kind of the human radiation exposure should be seriously reconsidered.

The radon level in water from different sources has been investigated randomly for different areas of Poland. Only in Lower Silesia, in the southwestern part of Poland, particularly in the Sudety Mountains, where elevated radon levels (up to 1,000 Bq/dm^3^) in groundwater occur, such measurements have been carried out methodically [12–14].

The aim of this study was to carry out a preliminary survey of radon levels in the underground water supplies located in the southern part of the Greater Poland region, in the Fore-Sudeten monocline tectonic unit. The main source of the drinking water in this area are the underground water supplies from quaternary, tertiary, cretaceous and jurassic geological formations. Using very good radon solubility in aromatic solvents, we previously successfully applied a direct extraction of radon from different kinds of groundwater samples for its precise and sensitive determinations [15–18].

## Experimental

### Water sampling

**Fig. 1:**
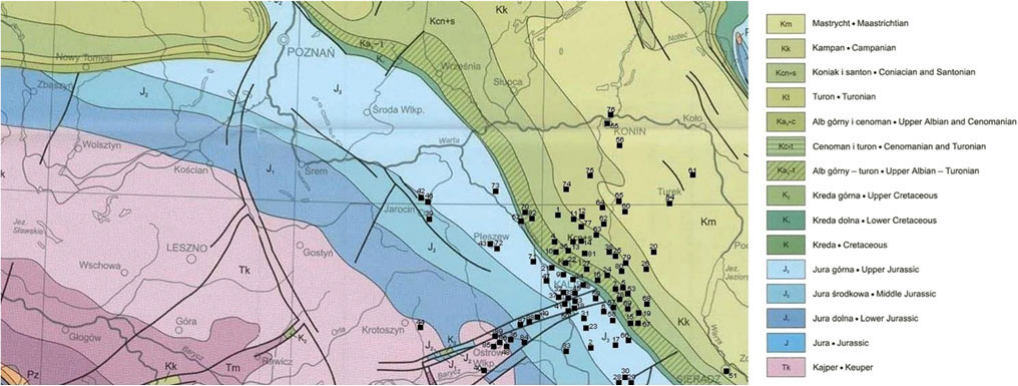
Geological formations of the underground water supplies [▪] in the Southern Greater Poland region

The overwhelming majority of the drinking water in the sampling area comes from ground water that is tapped by wells. The water samples were collected from various places, directly from underground supplies or the local water distribution networks as well as from domestic water taps in the area shown on the Fig. 1. Before filling the 1.5 dm^3^ plastic bottles, the water flowed for several minutes in order to collect the fresh water samples. The collected samples were transferred to the laboratory with delay time not exceeding 2 days.

### Method of measurements

**Fig. 2:**
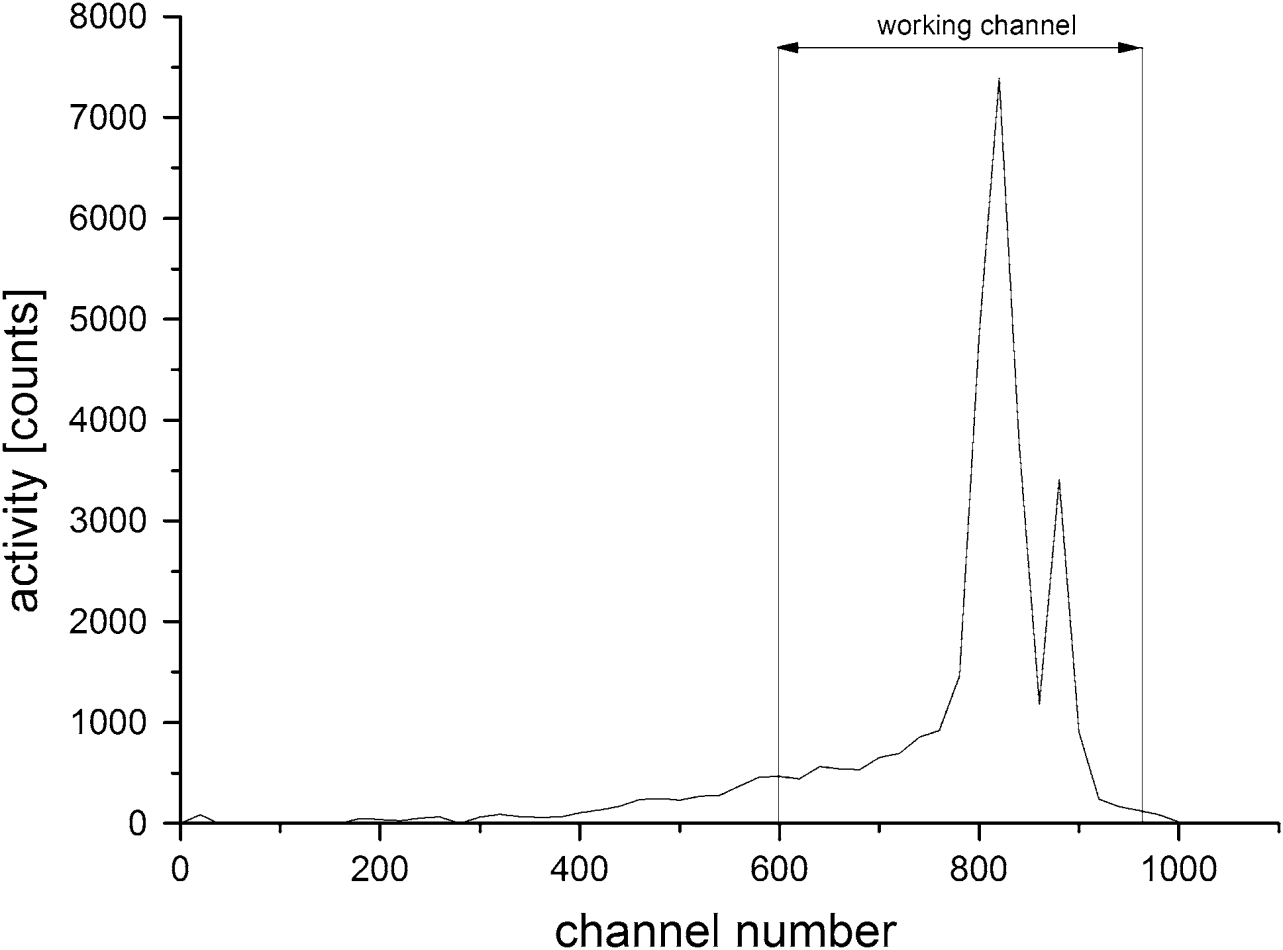
Spectrum of the Rn-222 and its progenies in the Beckman 3801 liquid scintillation counter

Two water samples (500 ml each) from each site were carefully transferred by laminar flow (to avoid radon escape) to two 0.5 dm^3^ glass flasks and 20 ml of liquid scintillation toluene based cocktail containing: 8 g/dm^3^ butyl-PBD and 0.3 g/dm^3^ dimethyl POPOP was added. After 5 min of vigorous shaking, the flasks were left for half an hour for complete separation of two liquid phases and after that time 18 ml of the upper scintillator phase with the extracted radon were taken directly to 20 ml glass scintillation vials. The activities of the eluted ^222^Rn and its four short-living daughters were measured (at least 3 h from the beginning of separation) in the fixed channel of the liquid scintillation counter Beckman 3801for 1 h for each sample. The spectrum of radon and its daughters is shown in the Fig. 2. Because of the lack of the α/β separation option in this device, an optimal counting channel has been chosen on the base of typical criterion e.g. *I* ^2^/*I*_b_ ratio, where *I* denotes the net activity of the sample and *I*_b_ background activity in cpm. The accuracy of the used analytical method has been checked by repeating whole procedure for the secondary standard of ^226^Ra solution (code 1R2), prepared by the Institute of Nuclear Chemistry and Technology in Warsaw, Poland for interlaboratory comparison studies. The certified concentration this radionuclide was equal to 1.955 ± 0.039 Bq/dm^3^. The standard ^222^Rn water solution from received plastic bottle was transferred in the identical method as in this work to 0.5 dm^3^ glass flask and was kept over 1 month with 20 ml of toluene based scintillation solution to ensure ^226^Ra–^222^Rn radioactive equilibrium. The obtained during this interlaboratory comparison procedure, value of ^222^Ra concentration was equal to 1.929 Bq/dm^3^, which confirms negligible loses of ^222^Rn during whole analytical procedure.

The average so-called calibration coefficient K of the method was calculated from the formula:

1 $$ K = C_{\text{Ra}} /I_{\text{s}} , $$

where *I*_s_ is the measured net activity of the standard in the chosen channel (cpm) and *C*_Rn_ is the ^222^Rn (activity of the standard calibration solution (Bq/dm^3^).

In these experiments the average value of calibration coefficient K = 0.0173 ± 0.0015 (Bq/dm^3^)/(imp/min) has been determined and used for calculation of radon concentration in routine sample measurements. The calculated relative standard deviation on the base of average activity of samples and background of counter was equal to 0.02 and total standard deviation of the whole method was appraised as equal to 0.1(10 %). The minimum detectable activity (MDA), which according to Currie estimates at the 95 % confidence level that the reported values are not subject of false positives, was calculated from the slightly modified formula [19]:

2 $$ {\text{MDA}} = \left( {2.71 + 4.66 \times \sqrt {\frac{B}{t}} } \right) \times K , $$

where *B* denotes blank in cpm, *t* is the standard time of counting and *K* is the calibration coefficient for this method.

Based on 0.5 dm^3^ sample volume and 60 min counting time, the estimated MDA value was equal to 0.11 Bq/dm^3^.

## Results and discussion

**Fig. 3:**
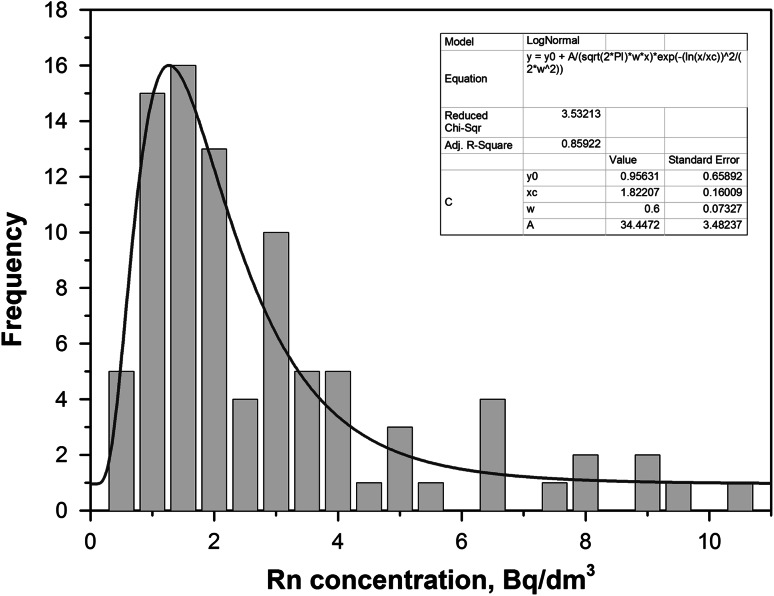
Log-normal distribution of the radon concentrations in drinking water samples

The distribution of radon concentrations for all measured water samples is shown in Fig. 3 and it well fits to typical log-normal distributions. The observed radon levels are relatively low: from 0.42 Bq/dm^3^ up to 10.52 Bq/dm^3^. The calculated arithmetic and geometric means of radon concentrations in the measured samples were equal to 2.67 and 1.92 Bq/dm^3^, respectively. The remaining radon concentration distribution parameters are shown in Table 1.

**Table 1:** Parameters of ^222^Rn concentration distribution in the Southern Greater Poland water

Parameter	
Number of samples	89
Arithmetic mean (Bq/dm^3^)	2.67
Arithmetic mean standard deviation (Bq/dm^3^)	2.27
Geometric mean (Bq/m^3^)	1.92
Log-normal distribution mean (Bq/m^3^)	1.70
Median (Bq/dm^3^)	1.78
Minimum concentration (Bq/dm^3^)	0.43
Maximum concentration (Bq/dm^3^)	10.52

It is worth noticing that the values of the geometric mean concentration, median and the mean of log-normal distributions are very close to each other and therefore, the geometric mean values should be taken for the effective dose calculations from water intake and inhalation of the escaping radon from water.

These results are very close to those obtained for water samples from: Polish Lowland [20], Central Poland [21], Mazowia [22] and Lesser Poland [23] as well as from the North-Eastern Poland regions [24].

As it is evident from Fig. 1 and Table 2. The underground drinking water supplies in the sampling area were drilled mostly in the Upper and Middle jurassic as well as in the Upper Albian-Turean or Coniacian and Santonian geological formations with low concentration of uranium and radium and consequently with low radon influx into existing water reservoirs. Low radon exhalation from such soils into the air has been also recently confirmed by us during the indoor radon concentration measurements in kindergartens and schools in this region [25]. The highest concentrations of ^222^Rn were found (Table 2) in the lower cretaceous and lower Jurasic formations. These observations are similar to those for the Central Poland region [21]. However, because of a few such underground water supplies in the examined region, only, the latest conclusion does not have sufficient statistical power.

**Table 2:** Radon concentrations in water from different geological formations of Southern Greater Poland Region

	Geological formation	Number of samples	Average ^222^Rn concentration (Bq/dm^3^)	Standard deviation (Bq/dm^3^)
1	Upper Jurassic	28	2.48	2.13
2	Middle Jurasic	7	3.00	2.78
3	Lower Jurasic	3	5.87	0.97
4	Campanian	7	1.79	0.83
5	Maastrichtian	8	1.86	1.39
6	Coniacian and Santonian	12	2.18	1.91
7	Upper Albian-Turean	17	2.87	2.58
8	Keuper	5	3.19	2.17
9	Lower Cretaceous	2	5.90	5.83

### Effective dose calculation

The total annual effective dose *E*_Rn_ for general population caused by occurrence of radon in drinking water and its domestic use is a sum of the effective doses due to radon ingestion with water −*E*_ing_ and inhalation from water-borne radon—*E*_inh_. Generally, the doses due to uranium series isotope ingestion with drinking water are negligibly low. Radiation exposures are predominantly caused by ^222^Rn, ^228^Ra, and ^210^Po radionuclides. Ingestion dose for particular radionuclide can be calculated from formula [26]:

3 $$ E_{\text{ing}} = {\text{ DCF}} \times A_{\text{ing}} , $$

where DCF (dose conversion factor) or dose coefficient is in Sv/Bq, which is connected with the effective dose due to ingestion of the unit activity of particular radionuclide, *A*_i_ is the total activity of ingested radionuclide in Bq.

In the case of radon in drinking water Eq. (3) can be modified:

4 $$ E_{\text{ing}} = {\text{ DCF }} \times \, A_{\text{Rn}} \times \, V_{\text{A}} , $$

where *A*_Rn_ is average radon activity in drinking water in Bq/dm^3^, *V*_A_ is estimated annual volume of water consumed directly from tap in dm^3^.

The dose coefficients depend on physicochemical properties of radionuclide, their accumulation and transfer between human organs and kinetics of excretion from human body. On the base such accepted biokinetic models describing the routes of intake and radionuclide behavior in human body, the International Committee for Radiological Protection (ICRP) has recommended the age depended values of the dose coefficient for ingestion for almost all natural and anthropogenic radionuclides (except of gaseous radon dissolved in water), which are also adopted by International Atomic Energy Agency (IAEA) and other international agencies, including EU (European Union) [26].

The most models for fate of radon ingested with water describe the radon as remaining in the stomach for several tens of minutes before being passed to the small intestine where it is transferred to blood and is rapidly lost from the body. Therefore, because of an effective self absorption of α-particles in the ingested water, only those from easily diffused Rn radionuclide can reach the stomach walls and contrary to inhalation of \Rn and its daughters, the dose from Rn, not from its progenies, to the stomach is determining factor for the total ingestion dose. The Commission on Life Sciences of the American National Research Council (NRC) approved the value of 3.5 × 10^−9^ Sv/Bq as an effective committed dose coefficient for radon ingestion [6]. However, later a more conservative value of 1 × 10^−8^ Sv/Bq has been also recommended [27].

There are also some controversies concerning human annual water intake. Since radon is readily lost from water by heating or boiling, the total annual water intake for so called “ICRP Standard Man” equals to 2 dm^3^ per day or 730 dm^3^ per year should not be taken into account for the dose calculation according to Eq. (4). More realistic value of 60 dm^3^ for the weighted direct annual consumption of tape water has been proposed in UNSCEAR 2000 Report (United Nations Scientific Committee on the Effects of Atomic Radiation) [28] and this value of *V*_A_ has been used in this work.

Therefore, for the average radon concentration of 1.92 Bq/dm^3^ the effective dose from water ingestion will be: *E*_ing_ = DCF × A_Rn _× *V*_A_ = 10^8 ^× 1.92 × 60 = 1.15 × 10^−6^ Sv or 1.15 μSv and for maximal observed radon concentration in water of 10.5 Bq.dm^3^ corresponding *E*_ing_ = 6.3 μSv. These doses in comparison with average effective dose from all natural sources ~2.4 mSv are really negligible.

The dose from inhalation of water-borne radon can be calculated from following formula:

5 $$ E_{\text{inh}} = {\text{ DCF }} \times \, A_{\text{Rn}} \times \, T \times \, F \times \, t, $$

where DCF is a radon dose conversion factor for radon inhalation DCF = 22 × 10^−9^ [Sv/(Bq h m^−3^], *A*_Rn_ is the average radon concentration in Bq/dm^3^, *T* is the radon transfer from water to air coefficient *T* = 0.1 dm^3^/m^3^. *t* is the average annual indoor occupancy in hours *t* = 7,000 h. *F* is the indoor radon—daughters equilibrium factor *F* = 0.4.

Introducing the above described values, one can obtain for average radon content in water:

$$ E_{\text{inh}} = 22 \times 10^{-9} \times 1.92 \times 0.1 \times 0.4 \times 7,000 = 11,827 \times 10^{-9} {\text{Sv or }}11.8\,{\upmu}\,{\text {Sv}},$$

and for maximal observed radon concentration in water of 10.5 Bq.dm^3^ corresponding *E*_inh_ = 64.7 μSv.

These doses due to inhalation of water-borne radon are one order higher of those from radon ingestion with water. Although they are still relatively low, one should take into account fact, that they are comparable and even higher of the annual effective dose caused by ingestion with food and water all remaining radionuclides from uranium and thorium series, which was for Central Poland population estimated as equal to 6 μSv, only [29].

Therefore, it should be clearly concluded, that despite of some uncertainties concerning the real values of the radon water to air transfer coefficient-*T* for any particular domestic conditions, not the ingestion of natural radionuclides with water but inhalation of the radon escaping from water is substantial part of radiological hazard due to presence of the natural radionuclides in drinking water. Ours observations are consistent with those concerning the radiological hazard from household water in southern Poland [30].
